# Supplementary material for: Bioactive Compounds Discovery from French Guiana Plant Extracts Through Antitubercular Screening and Molecular Networking
Source: Plants (Basel). 2025 Sep 30;14(19):3028. doi: 10.3390/plants14193028 (PMC12526154; doi:10.3390/plants14193028)
Supplement: Supplementary file 1 [file plants-14-03028-s001.zip › plants-3891435-supplementary.pdf]

plants
MDPI

Supplementary

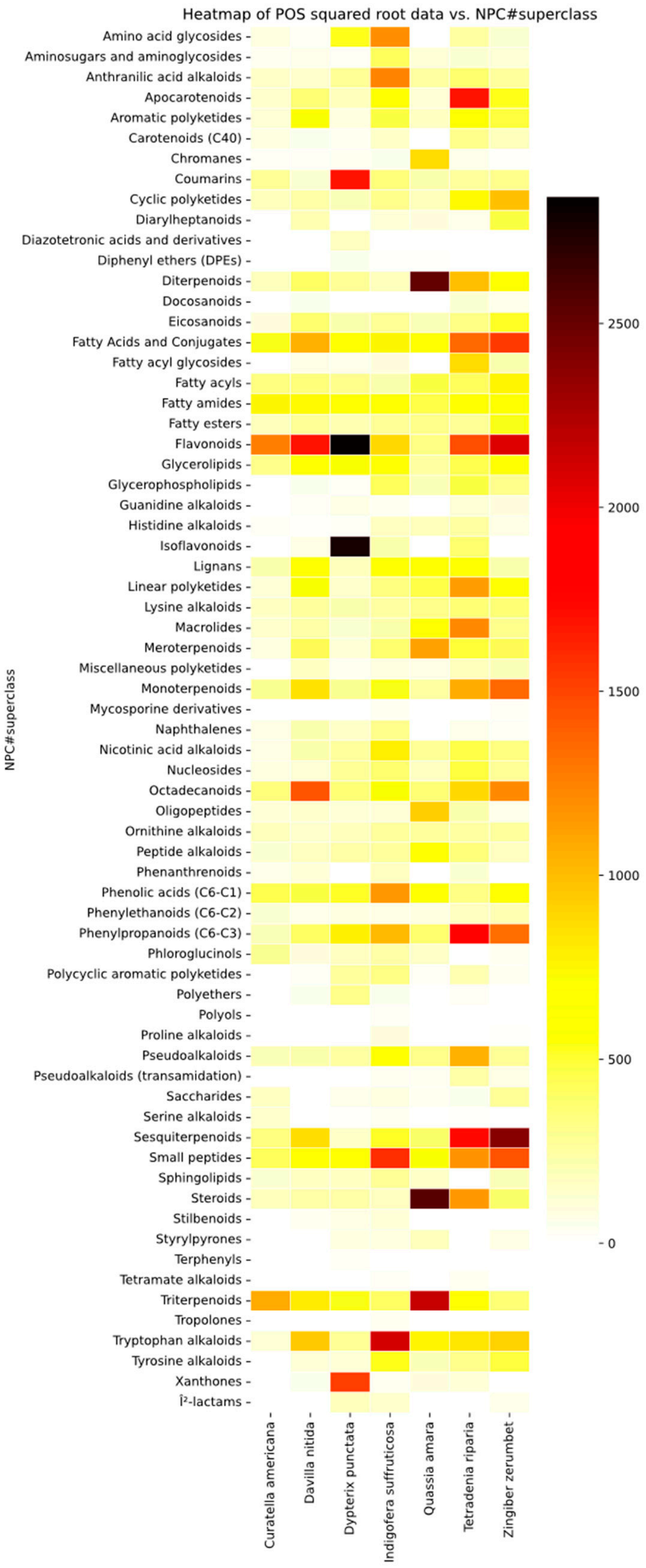

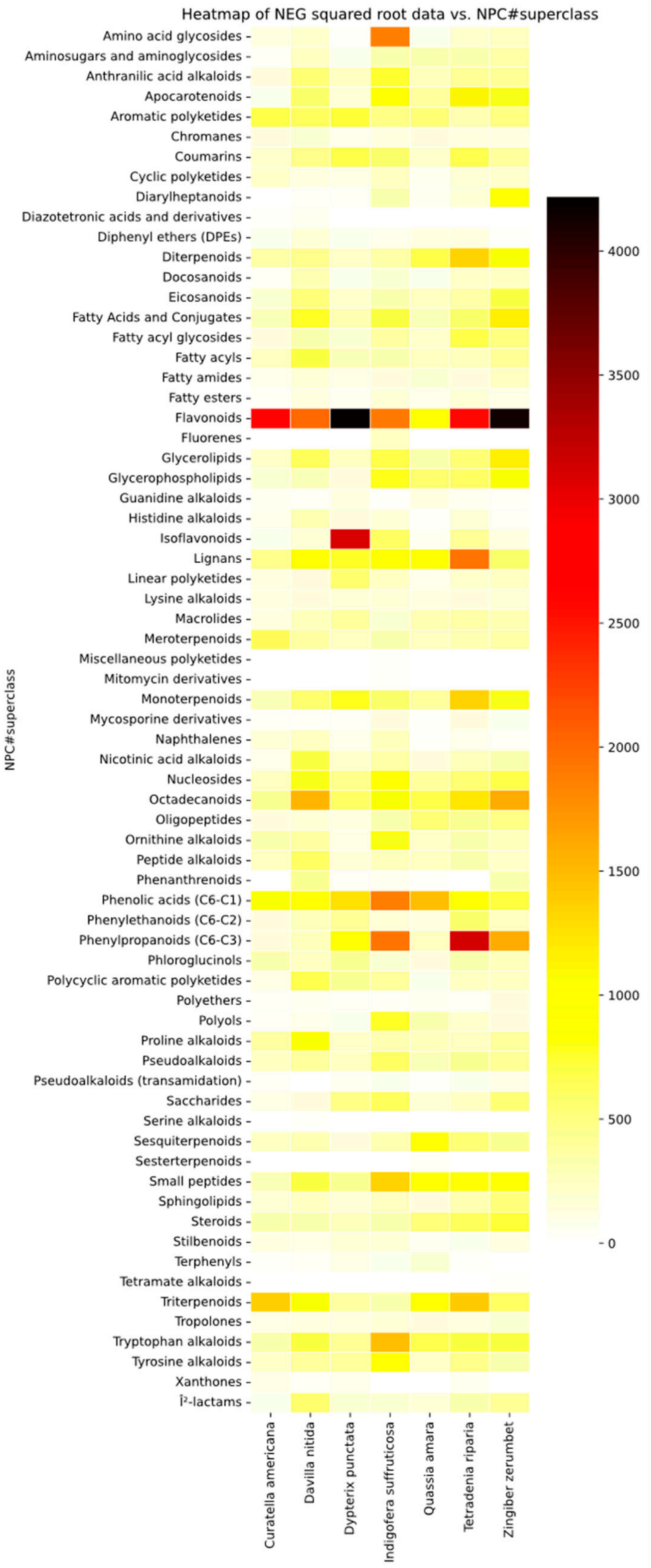

Figure S1. Heatmaps of the relative metabolite composition across species, according to metabolic superclasses.

Table S1. Extraction yields, for the UAE hydro-ethanolic crude extracts, and the decoction crude extracts.

Sample	Extraction yield ±SD
CA-B-E	20.3 ± 2.3
CA-B-D	8.8 ± 1.0
DN-L-E	29.4 ± 2.6
DN-L-D	25.3 ± 0.4
DP-B-E	25.4 ± 4.3
DP-B-D	8.4 ± 1.3
IS-L-E	19.0 ± 0.2
IS-L-D	22.0 ± 0.3
QA-W-E	16.01 ± 0.3
QA-W-D	14.5 ± 0.6
TR-L-E	14.1 ± 0.5
TR-L-D	18.5 ± 0.7
ZZ-L-E	13.7 ± 0.6
ZZ-L-D	12.2 ± 0.7
ZZ-R-E	19.9 ± 1.6
ZZ-R-D	10.04 10.0 ± 2.6

*SD = Standard deviation, calculated using Pearson's standard deviation method.

Table S2. Antitubercular activities of plant extracts against *Mtb* H37Ra.

Sample code	MIC_{50} [µg/mL]	MIC_{90} [µg/mL]
IS-L-E	>500	>500
IS-L-E-HE	244 ± 67	>500
IS-L-E-DM	147 ± 39	>500
IS-L-E-EA	>500	>500
IS-L-E-BU	>500	>500
IS-L-E-AQ	>500	>500
CA-B-E	>500	>500
CA-B-E-HE	>500	>500
CA-B-E-DM	>500	>500
CA-B-E-EA	>500	>500
CA-B-E-BU	>500	>500
CA-B-E-AQ	>500	>500
TR-L-E	>500	>500
TR-L-E-HE	128 ± 32	>500
TR-L-E-DM	178 ± 66	425 ± 130
TR-L-E-EA	>500	>500
TR-L-E-BU	>500	>500
TR-L-E-AQ	>500	>500
DN-L-E	>500	>500
DN-L-E-HE	>500	>500
DN-L-E-DM	>500	>500
DN-L-E-EA	>500	>500
DN-L-E-BU	>500	>500
DN-L-E-AQ	>500	>500
DP-B-E	>500	>500
DP-B-E-HE	>500	>500

DP-B-E-DM	>500	>500
DP-B-E-EA	>500	>500
DP-B-E-BU	>500	>500
DP-B-E-AQ	>500	>500
QA-W-E	>500	>500
QA-W-E-TA	>500	>500
QA-W-E-HE	>500	>500
QA-W-E-DM	>500	>500
QA-W-E-EA	>500	>500
QA-W-E-BU	>500	>500
QA-W-E-AQ	>500	>500
ZZ-L-E	32 ± 17	>500
ZZ-L-E-HE	211 ± 69	261 ± 96
ZZ-L-E-DM	>500	>500
ZZ-L-E-EA	>500	>500
ZZ-L-E-BU	>500	>500
ZZ-L-E-AQ	>500	>500
ZZ-R-E	>500	>500
ZZ-R-E-HE	130 ± 64	253 ± 166
ZZ-R-E-DM	74 ± 30	176 ± 39
ZZ-R-E-EA	>500	>500
ZZ-R-E-BU	>500	>500
ZZ-R-E-AQ	>500	>500
IS-L-D	>500	>500
IS-L-D-EA	166 ± 69	>500
IS-L-D-BU	>500	>500
CA-B-D	>500	>500
CA-B-D-EA	>500	>500
CA-B-D-BU	>500	>500
TR-L-D	>500	>500
TR-L-D-EA	>500	>500
TR-L-D-BU	>500	>500
DN-L-D	>500	>500
DN-L-D-EA	>500	>500
DN-L-D-BU	>500	>500
DP-B-D	>500	>500
DP-B-D-EA	>500	>500
DP-B-D-BU	>500	>500
QA-W-D	>500	>500
QA-W-D-EA	>500	>500
QA-W-D-BU	>500	>500
ZZ-L-D	>500	>500
ZZ-L-D-EA	>500	>500
ZZ-L-D-BU	>500	>500
ZZ-R-D	>500	>500
ZZ-R-D-EA	>500	>500
ZZ-R-D-BU	>500	>500

Table S3. Annotation of metabolites from the 72 samples by liquid chromatography quadrupole time-of-flight mass spectrometry (LC-Q-TOF-MS) analysis in positive and negative ion modes. Metabolites are sorted by retention times (RT).

Annotation	RT (min)	Molecular Formula	MS					MS/MS		IC	Ref
			Adducts	MM ID+	$[M+H]^+$ (Error in ppm)	MM ID-	$[M-H]^-$ (Error in ppm)	$[M+H]^+$ Fragments (relative intensity in %)	$[M-H]^-$ Fragments (relative intensity in %)		
Phenylalanin	0,77	$C_9H_{11}NO_2$	/	39	166.0864 (+0.9)	88	164.0716 (-0.6)	120.0810 (100); 103.0543 (54); 91.0543 (11); 79.0540 (7); 77.0383 (7); 107.0495 (5)	103.0557 (100); 147.0453 (80); 119.0495 (25); 96.9610 (31); 72.0106 (19)	L2a	[1]
Tryptophan	1,53	$C_{11}H_{12}N_2O_2$	$[M+Na]^+$ = 227.0792	236	205.0971 (-0.3)	511	203.0826 (+0)	146.0602 (100); 118.0652 (70); 188.0709 (56); 91.0541 (22); 159.0920 (17); 74.0233 (4)	116.0507 (100); 142.0665 (29); 74.0247 (10); 186.0597 (2)	L2a	[1]
Caprolactam (plastic pollution)	1,9	$C_6H_{11}NO$	$[M+Na]^+$ = 136.0735	306	114.0914 (+0.5)	/	N.D	114.0915 (100); 79.0540 (18); 96.0808 (17); 69.0694 (13); 55.0541 (11)	/	L2a	[2]
Zierin or isomer (Taxiphyllin)	2	$C_{14}H_{17}NO_7$	$[M+Na]^+$ = 334.0898	337	312.1080 (+0.7)	735	310.0933 (+0.2)	132.0446 (100); 150.0545 (7); 104.0491 (4)	160.0402 (100); 146.0241 (82); 179.0223 (54); 202.0507 (27); 89.0244 (27); 119.0348 (18)	L2b	[3]
Coronarian	2,67	$C_{12}H_{18}N_2O_{12}$	/	/	/	957	381.0789 (+0.5)	/	85.0287 (100); 113.0245 (80); 71.0139 (53); 174.9558 (36); 146.9618 (18); 125.0241 (18)	L2a	[1]

Indican	2,78	$C_{14}H_{17}NO_6$	$[M+Na]^+$ = 318.0950 $[M+FA-H]^-$ = 340.1036	464	296.1129 (+0.1)	996	294.0985 (+0.6)	134.0600 (100); 85.0284 (34); 146.0600 (24); 97.0289 (19); 242.0822 (11)	131.0378 (100); 174.0551 (5); 158.0617 (4); 144.0438 (4); 101.0257 (3); 85.0300 (2)	L2a	[4]
Epi-Catechin	3,16	$C_{15}H_{14}O_6$	$[M+Na]^+$ = 313.0680 $[2M+H]^+$ = 603.1475 $[2M-H]^-$ = 579.1513	551	291.0865 (+0.6)	1133	289.0719 (+0.5)	139.0392 (100); 123.0442 (66); 147.0440 (17); 207.0652 (15); 165.0546 (12); 179.0711 (4)	109.0296 (100); 123.0451 (97); 203.0715 (59); 245.0824 (54); 221.0820 (36); 137.0245 (36)	L2a	[1,5]
Caffeic acid	3,52	$C_9H_8O_4$	/	583	181.0497 (+0.9)	1240	179.0350 (+0.1)	163.0390 (100); 89.0386 (81); 135.0441 (68); 145.0285 (37); 117.0335 (34); 107.0492 (11)	135.0452 (100; 89.0395 (5); 107.0502 (3); 179.0353 (1)	L2a	[1]
Hemiphloin	4,38	$C_{21}H_{22}O_{10}$	/	709	435.1290 (+1)	1528	433.1139 (-0.3)	269.0808 (100); 107.0491 (72); 239.0702 (72); 297.0759 (72); 389.1230 (62); 223.0756 (43)	313.0716 (100); 343.0822 (51); 285.0765 (37); 123.0452 (23); 163.0400 (20); 151.0397 (19)	L2b	
Vanillin	5,19	$C_8H_8O_3$	/	796	153.0546 (-0.1)	1826	151.0401 (+0.2)	111.0443 (100); 65.0386 (42); 93.0331 (28)	108.0213 (100); 136.0155 (94); 79.0189 (25); 150.9999 (26); 262.9387 (7)	L2a	[6]
Procyanidin type B1	5,48	$C_{30}H_{26}O_{12}$	$[M+Na]^+$ = 601.1327 [M+Na-2H]- = 599 .1151	843	579.1504 (+1.2)	1938	577.1352 (+0.1)	127.0398 (100); 289.0720 (57); 139.0398 (58); 163.0396 (42); 271.0609 (35); 427.1025 (27)	289.0705 (100); 407.0757 (89); 125.0234 (55); 425.0861 (54); 577.1332 (26); 245.0812 (22)	L2b	[1]

p-coumaric acid	5,77	$C_9H_8O_3$	/	N.D	/	2100	163.04 (-0.4)	/	119.0502 (100); 93.0345 (13); 104.0266 (1)	L2a	[1]
(+) Catechin	5,95	$C_{15}H_{14}O_6$	$[M+Na+CO_2]^-$ = 357.0582	919	291.0865 (+0.6)	2195	289.0719 (+0.5)	139.0392 (100); 123.0444 (59); 147.0442 (20); 207.0659 (15); 165.0552 (12); 140.0428 (9)	109.0291 (100); 123.0445 (97); 245.0817 (55); 203.0710 (48); 137.0241 (37); 151.0393 (35)	L2a	[1]
5,7-dihydroxy-2-(3,4,5-trihydroxyphenyl)-3,4-dihydro-2h-1-benzopyran-3-yl 3,4,5-trihydroxybenzoate	6,18	$C_{22}H_{18}O_{11}$	$[M+Na]^+$ = 479.0598	985	459.0927 (+1.1)	2322	457.0775 (-0.3)	139.0394 (100); 151.0393 (13); 153.0187 (13); 289.0707 (6); 163.0397 (4); 181.0500 (3)	169.0139 (100); 125.0242 (42); 305.0661 (8); 161.0248 (4); 137.0243 (3); 287.0603 (2)	L2a	[1]
Ethyl gallate	6,2	$C_9H_{10}O_5$	/	N.D	/	2335	197.0456 (+0.3)	/	125.0244 (100); 169.0141 (48); 197.0457 (44); 140.0116 (41); 111.0090 (23); 106.0061 (8)	L2a	[1,5]
Roseoside	6,31	$C_{19}H_{30}O_8$	$[M+Na]^+$ = 409.1841 $[M+Cl]^-$ = 421.1634	1014	387.2016 (+0.7)	2386	385.1866 (-0.5)	207.1385 (100); 95.0859 (67); 123.0811 (45); 149.0967 (33); 189.1282 (20); 113.0602 (18);	153.0918 (74); 161.0453 (37); 205.1233 (31); 223.1343 (29);	L2a	[7]
Isatin	6,54	$C_8H_5NO_2$	$[M+Na]^+$ = 166.0502	1068	148.0393 (+0)	/	N.D	120.044 (99); 92.0497 (72); 97.0077 (27); 65.0381 (15); 130.0292 (14)	/	L2a	[8]
Tuberonic acid	6,73	$C_{12}H_{18}O_4$	$[M+Na]^+$ = 249.1099	1109	227.1281 (+1.4)	2617	225.1132 (-0.2)	131.0857 (100); 149.0963 (95); 85.0648 (59);	59.0145 (100); 89.0239 (7); 225.110 (3)	L2a	[6]

								191.1067 (53); 107.0857 (47); 91.0545 (46)			
Indole-3-carboxyaldehyde	7,49	C_9H_7NO	$[M+Na]^+$ = 168.0422	1320	146.0601 (+0.4)	3188	144.0454 (-0.6)	118.0654 (100); 91.0545 (53); 146.0602 (8)	144.0454 (100); 126.0352 (10); 116.0503 (9); 114.0348 (6); 115.0420 (5); 142.0299 (3)	L2a	[9]
Isololiolide	7,5	$C_{11}H_{16}O_3$	$[M+Na]^+$ = 219.0993	1324	197.1174 (+0.9)	/	N.D	133.1018 (100); 107.0862 (76); 105.0699 (68); 179.1067 (63); 91.0544 (54); 161.0958 (48)	/	L2a	[7]
3-O-galloyl(epi)catechin-(epi)catechin	7,67	$C_{37}H_{30}O_{16}$	$[M+Na]^+$ = 753.1437 $[M+Na-2H]^-$ = 751.1256	1368	731.1615 (+1.1)	3317	729.1463 (+0.3)	127.0393 (100); 289.0714 (71); 409.0922 (42); 247.0610 (48); 275.0553 (47); 63.0391 (44)	407.0755 (100); 289.0706 (89); 125.0236 (35); 441.0811 (28); 577.1332 (20); 169.0131 (18); 451.1007 (22)	L2a	[5]
Deacetylboronolide	8,1	$C_{12}H_{20}O_5$	$[M+Na]^+$ = 267.1203	1494	245.1387 (+1.4)	3630	243.1237 (-0.4)	97.0286 (100); 111.0445 (29); 83.0488 (28); 95.0494 (27); 145.1009 (21); 99.0808 (24)	130.9666 (100); 118.9662 (95); 174.9550 (68); 146.9611 (53); 161.9499 (45); 102.9709 (40)	L2b	SIRIUS
Chicoric acid	8,25	$C_{22}H_{18}O_{12}$	/	/	N.D	3806	473.0724 (-0.3)	/	179.0351 (100); 149.0092 (85); 135.0453 (37); 311.0406 (20); 293.0302 (14); 219.0298 (13)	L2a	[1]
Loliolide	8,27	$C_{11}H_{16}O_3$	$[M+Na]^+$ = 219.0995	1549	197.1173 (+0.4)	/	N.D	179.1072 (100); 133.1017 (76); 107.0861	/	L1	Standard

								(61); 105.0703 (50); 91.0544 (45); 161.0966 (43)			injection
Kaempferol 3-Rhamnosyl-(1->6)-Glucosyl-(1->6)-Galactoside or isomer	8,51	$C_{33}H_{40}O_{20}$	$[M+Na]^+$ = 779.1997	1652	757.2197 (+1.5)	3999	755.2044 (+0.5)	287.0558 (100); 129.0555 (9); 449.1093 (5); 147.0661 (6); 85.0287 (5)	284.0332 (74); 285.0399 (35) (aglycone)	L2a	[10]
Coumarine	8,73	$C_9H_6O_2$	/	1705	147.0442 (+1)	/	N.D	147.0434 (100); 91.0538 (91); 103.0540 (80)	/	L1	Standard injection
Quercetin 3-(apiosylrutinoside) or isomer	8,78	$C_{32}H_{38}O_{20}$	$[M+Na]^+$ = 765.1868	1731	743.2041 (+1.6)	4216	741.1880 (-0.5)	303.0509 (100); 465.1043 (6); 129.0552 (6); 85.0291 (3)	300.0277 (76) (aglycone); 178.9986 (4)	L2a	[1]
Catechin-O-gallate	8,93	$C_{22}H_{18}O_{10}$	$[M+Na]^+$ = 465.0803 $[M+Na-2H]^-$ = 463.0640	1784	443.0973 (+0.1)	4342	441.0828 (+0.2)	123.0441 (100); 139.0390 (41); 153.0182 (19); 165.0545 (3); 273.0761 (4)	169.0136 (100); 289.0711 (56); 125.0240 (48); 245.0813 (11); 203.0709 (7); 109.0290 (6)	L2a	[11]
Myricetin-3-Rhamnoside	9,1	$C_{21}H_{20}O_{12}$	$[M+Na]^+$ = 487.0859	1853	465.1031 (+0.7)	4472	463.0884 (+0.4)	319.0456 (100); 85.0287 (21); 129.0555 (15); 71.0496 (5); 147.0670 (3)	316.0222 (100); 463.0879 (53); 271.0244 (21); 287.0193 (12); 178.9983 (4); 259.0246 (3)	L2a	[1]
Ditolylguanidine	9,13	$C_{15}H_{17}N_3$	/	1867	240.1495 (-0.1)	/	N.D	133.0763 (48); 108.0812 (44); 106.0653 (43); 91.0542 (16); 116.0497 (15); 223.1218 (12)	/	L2a	[12]

Hyperoside	9,24	$C_{21}H_{20}O_{12}$	/	1906	465.1033 (+1.2)	4603	463.0880 (-0.4)	303.0505 (100); 91.0398 (6); 85.0284 (4); 97.0287 (3); 185.1148 (2); 145.0533 (1)	300.0281 (100); 463.0889 (51); 271.0246 (21); 255.0297 (10); 151.0037 (4); 243.0317 (3);	L1	Standard injection
Quercetin-3-O-beta-glucuronide	9,36	$C_{21}H_{18}O_{13}$	/	1949	479.0827 (+1.4)	4710	477.0676 (+0.3)	303.0509 (100); 113.0237 (5); 85.0287 (4); 131.0340 (3); 159.0295 (3); 141.0183 (2)	301.0358 (100); 151.0040 (10); 178.9988 (6); 283.0247 (3); 245.0462 (2); 273.0399 (2)	L2a	[1]
Rutin	9,42	$C_{27}H_{30}O_{16}$	/	1969	611.1614 (+1.2)	4769	609.1460 (-0.2)	303.0511 (100) (aglycone); 465.1041 (4); 85.0289 (7); 129.0555 (6); 147.0662 (3)	300.0278 (100) (aglycone) ; 271.0250 (3); 255.0308 (1); 151.0039 (2); 178.9987 (2)	L1	Standard injection
Lyoniresinol	9,44	$C_{22}H_{28}O_8$	$[M+Na]^+$ = 443.1679	1986	421.1856 (-0.2)	4791	419.1713 (0.4)	249.1133 ; 267.1233; 167.0691 (5);	327.1239 (100); 151.0399 (23); 312.0998 (20); 357.1337 (18); 136.0150 (12); 345.1348 (8)	L2a	[13]
7-O-Methyl-quercetin-3-O-galactoside-6''-rhamnoside	9,48	$C_{34}H_{42}O_{20}$	/	2019	771.2351 (+1.1)	4824	769.2196 (-0.1)	317.0665 (100); 129.0549 (12); 147.0658 (7); 85.0287 (7); 293.1239 (3); 239.0916 (2)	769.2192 (100); 314.0432 (63); 299.0205 (3)	L2a	[1]
Quercetin-3-O-glucoside	9,56	$C_{21}H_{20}O_{12}$	$[M+Na]^+$ = 487.0849	2032	465.1033 (+1.2)	4897	463.088 (-0.4)	303.0499 (100); 145.0494 (5); 85.0283 (5); 127.0391 (3); 97.0281 (3)	30.0284 (100); 463.0889 (47); 271.0258 (24); 255.0306 (12);	L2a	[14]

									243.0306 (4); 151.0045 (3)		
Camelliaside B (Kaempferol 3-(2G-Xylosyl-rutinoside)	9,68	$C_{32}H_{38}O_{19}$	/	2085	727.2093 (+1.8)	5001	725.1931 (-0.5)	287.0562 (100); 449.1092 (5); 129.0551 (6); 85.0293 (4); 243.0864 (3)	284.0325 (91); 178.9995 (3); 112.9853 (3)	L2b	
Carinol	9,71	$C_{20}H_{26}O_7$	/	N.D	/	5030	377.1612 (+1.6)	/	149.0240 (100); 329.1393 (86); 165.0558 (28); 174.9561 (26); 314.1133 (25); 195.0654 (14)	L2b	
Procyanidin type A2	9,72	$C_{30}H_{24}O_{12}$	/	N.D	/	5049	575. 1195 (+0)	/	285.0393 (100); 289.0705 (61); 575.1182 (52); 449.0885 (43); 423.0724 (34); 407.0749 (30)	L2a	[1]
Luteolin-7-O-glucoside	9,81	$C_{21}H_{20}O_{11}$	/	2131	449.1081 (+0.6)	5129	447.0933 (+0)	287.0550 (100); 153.0178 (1)	285.0409 (100); 447.0938 (72); 174.9567 (6)	L2a	[15]
Kaempferol-3-O-rutinoside	10	$C_{27}H_{30}O_{15}$	/	2216	595.1655 (-0.4)	5288	593.1512 (+0)	287.0550 (100); 85.0282 (12); 129.0541 (7); 449.1133 (3); 289.0633 (3); 147.0641 (2)	593.1524 (100); 284.0321 (52); 112.9862 (29); 174.9567 (17); 388.8680 (15); 476.8485 (13)	L2a	[6]
1,2-dideacetyl-boronolide	10,01	$C_{14}H_{22}O_6$	$[M+NH_4]^+$ = 304.1636 $[M+Na]^+$ = 309.1309	2218	287.1492 (+1)	/	N.D	191.1067 (100); 163.1123 (55); 209.1179 (49); 145.1021 (42); 173.0969 (14); 149.0606 (14)	/	L2a	[16]

Quercetin-3-O-pentoside	10,16	$C_{20}H_{18}O_{11}$	/	2267	435.0922 (+0)	5416	433.0775 (-0.3)	303.0505 (100); 73.0283 (8); 115.0393 (2)	300.0274 (100); 433.0781 (36); 271/0249 (26); 255.0291 (11); 243.0306 (4); 151.0051 (2)	L2a	[1]
Maesopsin	10,21	$C_{15}H_{12}O_6$	$[M+Na+CO_2]^-$ = 355.0417	2281	289.0705 (-0.6)	5461	287.0562 (+0.3)	153.0181 (100); 149.0227 (76); 107.0494 (67); 215.0698 (55); 243.0653 (55); 197.0591 (10)	125.0243 (100); 259.0610 (60); 177.0558 (26); 151.0035 (18); 243.0657 (17); 201.0554 (14)	L2a	[1]
Quercetin-3-O-hexoside	10,22	$C_{21}H_{20}O_{12}$	/	2289	465.103 (+0.5)	5463	463.0882 (+0)	303.0509 (100); 465.1024 (21); 177.0554 (17); 319.0453 (16); 153.0202 (5); 85.0277 (4)	301.0337 (100); 463.0855 (63); 174.9571 (29); 146.9605 (21); 317.0275 (13); 534.7611 (9)	L2a	[1]
Quercetin-3-(6''-malonyl)-glucoside	10,32	$C_{24}H_{22}O_{15}$	$[M+Na]^+$ = 573.0874 $[M+Na-2H]^-$ = 571.0705	2321	551.1042 (+1.9)	5554	549.0880 (-1.1)	303.0509 (100); 127.0400 (11); 231.0510 (3); 145.0512 (7); 159.0296 (6); 109.0291 (7)	300.0270 (100); 505.0983 (60); 271.0240 (9); 255.0284 (3); 151.0040 (3)	L2a	[17]
Penipanoid C	10,51	$C_{15}H_{10}N_2O_3$	/	2359	267.0765 (+0.3)	/	N.D	120.0444 (63); 249.0648 (46); 130.0287 (26); 239.0815 (17); 146.0233 (13); 92.0496 (13)	/	L2b	SIRIUS
3-O-Neohesperidoside Kaempferol	10,61	$C_{27}H_{30}O_{15}$	$[M+Na]^+$ = 617.1498	2402	595.1665 (+1.3)	5814	593.1510 (-0.3)	287.0559 (100); 449.1072 (4); 129.0556 (7); 85.0290 (8)	285.0401 (100); 255.0301 (5); 227.0369 (3); 327.0529 (2); 175.0385 (1)	L2a	[18]

Cyperenol or derivative	10,64	$C_{15}H_{24}O$	[M-H2O+H]+ = 203.1787	2417	221.1905 (+2.3)	/	N.D	147.1174 (100); 95.0856 (89); 105.0701 (87); 203.1796 (56); 119.0859 (53); 81.0694 (47)	/	L3	
Glycosylated Quercetin	10,67	$C_{23}H_{20}O_{14}$	/	2438	521.0932 (+1.2)	5856	519.0781 (+0.1)	303.0506 (100); 159.0291 (9); 219.0502 (6); 113.0237 (6); 85.0282 (6); 173.04489 (6)	301.0354 (100); 415.0677 (25); 151.0040 (8); 178.9985 (6); 255.0298 (4); 271.0249 (4)	L2b	
Quercitrin	10,82	$C_{21}H_{20}O_{11}$	/	2511	449.1085 (+1.5)	5993	447.0932 (-0.2)	303.0498 (100); 85.0277 (25); 129.0543 (20); 71.0489 (8); 147.0653 (4); 101.0597 (1)	300.0280 (100); 447.0939 (52); 271.0253 (31); 255.0311 (12); 151.0039 (8); 243.0317 (6)	L2a	[1]
(4r)-3,5,5-trimethyl-4-[(1e,3r)-3-{[(2r,3r,4s,5r,6r)-3,4,5-trihydroxy-6-(hydroxymethyl)oxan-2-yl]oxy}but-1-en-1-yl]cyclohex-2-en-1-one : Byzantionoside B with an insaturation	10,99	$C_{19}H_{30}O_{7}$	$[M+Na]^+$ = 393.1894 $[M+Cl]^-$ = 405.1685	2583	371.2070 (+1.5)	/	N.D	151.1132 (100); 209.1542 (95); 191.1437 (84); 121.1024 (25); 173.1326 (20); 107.0853 (20)	/	L2b	
Xanthone	11,03	$C_{29}H_{29}NO_{12}$	/	2598	584.1772 (+1.6)	6184	582.1613 (-0.7)	289.0703 (100); 270.0758 (39); 139.0389 (35); 127.0389 (32);	279.0538 (100); 430.1143 (45); 268.0617 (36); 125.0249 (15);	L2b	SIRIUS

								163.03886 (25); 422.137 (15)	402.0978 (10); 252.0670 (16)		
Isorhamnetin 3-O-rutinoside (narcissin)	11,04	$C_{28}H_{32}O_{16}$	/	2606	625.1772 (+1.4)	6189	623.1614 (-0.6)	317.0660 (100); 129.0547 (6); 85.0279 (6); 479.1209 (4); 147.0652 (4); 319.0707 (3)	315.0505 (100); 623.1612 (85); 299.0197 (9); 300.0270 (8)	L1	Standard injection
Apigenin-7-O-glucoside	11,1	$C_{21}H_{20}O_{10}$	/	2634	433.1131 (+0.4)	6244	431.0986 (+0.5)	271.0589 (100); 272.0628 (18)153.0188 (1)	268.0378 (100); 431.0980 (76)	L2a	[1]
Narcissin isomer	11,18	$C_{28}H_{32}O_{16}$	/	2656	625.1776 (+2.1)	6315	623.1610 (-1.2)	317.0661 (100); 479.1194 (3); 129.0550 (6); 85.0284 (9); 147.0653 (4)	315.0506 (100); 299.0198 (8)	L2b	
Byzantionoside B or isomer	11,38	$C_{19}H_{32}O_7$	$[M+Na]^+$ = 395.2053	2757	373.2223 (+0.6)	6510	371.2074 (-0.3)	211.1700 (100); 135.1173 (57); 109.1017 (46); 193.1596 (41); 175.1487 (24); 119.0863 (14)	174.9561 (100); 99.0079 (28); 130.9659 (22); 166.9239 (22); 304.9147 (15); 234.8722 (17)	L2a	[1]
Rosmarinic acid	11,55	$C_{18}H_{16}O_8$	$[M+Na-2H]^-$ = 381.0598	2819	361.0919 (+0.3)	6664	359.0774 (+0.4)	163.0388 (100); 135.0437 (17); 139.0388 (10); 145.0282 (8); 181.0495 (7); 117.0337 (5)	161.0240 (100); 197.0541 (37); 135.0448 (27); 133.0291 (26); 179.0346 (21); 123.0447 (13)	L2a	[6]
Luteolin 7-(6-acetylglucoside)	11,81	$C_{23}H_{22}O_{12}$	/	/	N.D	6916	489.1037 (-0.3)	/	312.0272 (100); 327.0507 (46); 489.1034 (35); 313.0307 (21); 283.0244 (4); 474.0784 (2)	L2b	

Hydroxycortisol derivative	11,92	$C_{21}H_{30}O_6$	$[M+Na]^+$ = 401.1937 $[2M+Na]^+$ = 779.3997	3003	379.2116 (+0.2)	6981	377.1971 (+0.4)	343.1907 (100); 121.0646 (88); 153.0907 (76); 283.1694 (54); 223.1480 (46); 361.2011 (31)	174.9559 (100); 377.1974 (80); 362.1747 (52); 347.1504 (27); 146.9621 (23); 394.1661 (22)	L2b	[19]
Haploside D	11,93	$C_{30}H_{34}O_{18}$	/	/	N.D	7001	681.1672 (-0.1)	/	341.0659 (100); 326.0422 (24); 311.0190 (23); 293.0871 (3)	L2a	[1]
Afzelin	12,26	$C_{21}H_{20}O_{10}$	$[M+Na+CO_2]^-$ = 499.0860	3162	433.1135 (+1.3)	7317	431.0983 (-0.2)	287.0545 (100); 85.0276 (21); 129.0534 (16); 71.0480 (10); 147.0654 (5)	284.0328 (100); 255.0298 (56); 227.0354 (42); 187.0413 (1)	L2a	[1]
7,4'-Dihydroxflavone	12,3	$C_{15}H_{10}O_4$	/	3185	255.0656 (+1.6)	7352	253.0507 (+0.3)	255.0641 (100); 145.0282 (18); 137.0238 (12); 119.0501 (8); 91.0547 (5)	117.0346 (100); 253.0504 (91); 135.0088 (17); 91.0187 (10); 208.0516 (3); 224.0484 (3)	L2a	[1]
Byzantionoside B with an insaturation + malonic acid (--> 3-oxo-3-[(3,4,5-trihydroxy-6-{[4-(2,6,6-trimethyl-4-oxocyclohex-2-en-1-yl)butan-2-yl]oxy}oxan-2-yl)methoxy]propanoic	12,51	$C_{22}H_{32}O_{10}$	$[M+Na]^+$ = 479.1896	3276	457.2075 (+1.5)	/	N.D	209.1544 (100); 151.1121 (38); 165.1275 (12); 137.0960 (9); 193.1602 (8); 127.0397 (7)	/	L2b	

acid with an insaturation)											
6-Methoxyluteolin	12,59	$C_{16}H_{12}O_7$	/	3310	317.0658 (+0.7)	7571	315.0510 (-0.1)	317.0643 (10); 302.0421 (37); 168.0050 (11); 186.0151 (11); 137.0240 (4); 68.9949 (2)	300.0276 (100); 315.0517 (51); 136.9877 (10); 133.0295 (10); 201.0192 (8); 199.0395 (6)	L2a	[1]
3-phenyl-2-[(2S,3R,4S,5S,6R)-3,4,5-trihydroxy-6-[[(E)-3-(4-hydroxyphenyl)prop-2-enoyl]oxymethyl]oxan-2-yl]oxyprop-2-enoic acid	12,63	$C_{24}H_{24}O_{10}$	/	/	N.D	7626	471.1302 (+1.1)	/	163.0390 (100); 145.0287 (77); 119.0494 (65); 307.0811 (54); 285.0406 (3); 447.0907 (2)	L2a	[1]
Eriodictyol	12,72	$C_{15}H_{12}O_6$	$[M+Na-2H]^-$ = 309.0390	3363	289.0707 (+0.1)	7695	287.0561 (+0)	153.0178 (100); 163.0384 (59); 289.0701 (50); 135.0439 (20); 145.0282 (7); 89.0381 (5)	135.0456 (100); 151.0042 (42); 107.0139 (8); 83.0154 (3); 117.0355 (2); 89.0403 (1)	L2a	[20]
Ehletianol C	12,76	$C_{30}H_{36}O_{10}$	/	/	N.D	7726	555.2247 (+2)	/	525.2135 (100); 343.1199 (66); 181.0867 (56); 166.0629 (45); 298.0849 (27); 537.2093 (22)	L2b	SIRIUS

Hydroxycortisol isomer	12,92	$C_{21}H_{30}O_6$	$[M+Na]^+$ = 401.1935	3463	379.2118 (+0.7)	7859	377.1970 (+0.1)	361.2009 (46); 343.1912 (40); 225.1271 (13); 127.0752 (12); 251.1429 (12); 151.0751 (12)	174.9562 (100); 146.9620 (26); 242.9442 (13); 362.1704 (13); 377.2014 (12); 112.9851 (11)	L2b	
Byzantionoside B with an insaturation + malonic acid	12,97	$C_{22}H_{32}O_{10}$	$[M+Na]^+$ = 479.1898	3482	457.2076 (+1.7)	/	N.D	209.1551 (100); 191.1434 (44); 151.1124 (39); 120.0452 (24); 137.0967 (22); 160.0406 (23); 107.0863 (14)	/	L2b	
Byzantionoside B or isomer + malonic acid (3-oxo-3-[(3,4,5-trihydroxy-6-{[4-(2,6,6-trimethyl-4-oxocyclohex-2-en-1-yl)butan-2-yl]oxy}oxan-2-yl)methoxy]propanoic acid)	13,39	$C_{22}H_{34}O_{10}$	$[M+Na]^+$ = 481.2047	3611	459.2231 (+1.4)	/	N.D	211.1701 (100); 193.1596 (24); 135.1177 (22); 109.1020 (17); 175.1490 (13); 95.0861 (5)	/	L2b	
6-beta-Hydroxycortisol or isomer	13,43	$C_{21}H_{30}O_6$	$[M+Na]^+$ = 401.1937	3628	379.2117 (+0.5)	8223	377.1972 (+0.6)	361.2009 (100); 343.1904 (99); 267.1370(17); 325.1789 (15); 239.1418 (15); 165.0909 (12)	174.9573 (100); 360.1602 (32); 377.1951 (23); 333.1708 (22); 327.1241 (18); 104.9557 (13)	L2a	[19]
Quercetin	13,58	$C_{15}H_{10}O_7$	/	3678	303.0495 (-1.4)	8373	301.0354 (+0.1)	153.0193 (31); 257.0442 (24); 240.9116 (21); 193.0051 (18); 279.9207 (15)	151.0037 (100); 301.0365 (73); 178.9991 (47); 121.0296 (31);	L1	Standard injection

									107.0140 (24); 83.0143 (9)		
Luteolin	13,71	$C_{15}H_{10}O_6$	/	3724	287.0552 (+0.6)	8476	285.0405 (+0.1)	287.0543 (100); 153.0171 (18); 135.0446 (14); 167.0232 (7); 84.9587 (6); 137.0242 (4)	285.0398 (100); 133.0291 (51); 151.0027 (10); 175.0396 (5); 107.0138 (4); 199.0402 (3)	L1	Standard injection
Diterpenoid	13,79	$C_{28}H_{40}O_{11}$	$[M+Na]^+$ = 575.2478	3756	553.2650 (+1.2)	/	N.D	373.2020 (100); 207.1019 (47); 329.1755 (17); 221.1177 (13); 193.1227 (10); 233.1176 (9)	/	L3	
Sesquiterpenoid	14,39	$C_{15}H_{24}O$	$[M-H_2O+H]^+$ = 203.1787	3948	221.1901 (+0.5)	/	N.D	109.1008 (100); 95.0850 (85); 147.1161 (54); 203.1787 (50); 119.0845 (48); 91.0539 (43)	/	L3	
Hispidulin isomer	14,44	$C_{16}H_{12}O_6$	$[M+Na]^+$ = 323.0528	3965	301.0713 (+2.1)	9028	299.0561 (+0)	286.0473 (33); 168.0059 (20); 186.0168 (13); 121.0293 (3); 119.0509 (3); 127.0023 (2)	284.0330 (100); 136.9887 (13); 117.0344 (6); 183.0455 (5); 212.0481 (5); 227.0356 (4)	L2a	[1]
Nigakilactone B	14,49	$C_{22}H_{32}O_6$	$[M+Na]^+$ = 415.2094	3988	393.2276 (+1.1)	9051	391.2129 (+0.7)	343.1908 (100); 375.2172 (56); 251.1429 (39); 223.1477 (38); 283.1693 (35); 265.1589 (27)	391.2139 (100); 376.1895 (73); 408.1810 (30); 361.1652 (29); 267.1372 (10); 297.1804 (6)	L2b	
[(4E)-7-acetyloxy-6-hydroxy-2-methyl-10-oxo-2,3,6,7,8,9-	14,52	$C_{24}H_{24}O_{10}$	/	/	N.D	9085	471.1296 (-0.2)	/	145.0298 (100); 163.0401 (18); 307.0822 (16); 117.0347 (12);	L2a	[1]

hexahydroox-ecin-3-yl] (E)-but-2-enoate									265.0713 (10); 205.0501 (7)		
Afzelin acetate	14,66	$C_{23}H_{22}O_{11}$	$[M+Na]^+$ = 497.1048 $[M+Na+CO_2]^-$ = 541.0967	4056	475.1242 (+1.5)	9183	473.1087 (-0.5)	189.0750 (100); 129.0540 (88); 287.0543 (80); 111.0436 (44); 171.0648 (29); 71.0486 (9)	284.0327 (100); 255.0303 (34); 227.0354 (23)	L2b	[1]
Eupatorin or isomer	14,79	$C_{18}H_{16}O_7$	/	4117	345.0971 (+0.6)	9280	343.0824 (+0.2)	345.0969 (100); 287.0544 (48); 312.0626 (32); 315.0494 (29); 330.0730 (12); 297.0397 (10)	313.0360 (100); 298.0125 (74); 270.0174 (47); 328.0595 (40); 136.9879 (29); 343.0831 (19)	L2a	[6]
Naringenin	14,95	$C_{15}H_{12}O_5$	$[M+Na+CO_2]^-$ = 339.0492	4187	273.0758 (+0.2)	9393	271.0613 (+0.4)	153.0190 (100); 147.0449 (45); 119.0498 (21); 91.0548 (9); 123.0449 (5); 171.0294 (4)	119.0500 (100); 151.0035 (72); 107.0135 (17); 93.0342 (9); 177.0192 (8); 83.0137 (7)	L2a	[1]
Nigakilactone B derivative	15,05	$C_{22}H_{34}O_6$	$[M+Na]^+$ = 417.2253	4228	395.2431 (+0.7)	/	N.D	327.1956 (100); 377.2325 (70); 345.2068 (51); 315.1957 (43); 359.2215 (38); 283.1694 (32)	/	L3	
Jaceosidin	15,22	$C_{17}H_{14}O_7$	$[M+Na]^+$ = 353.0643	4285	331.0813 (+0.2)	9561	329.0664 (-0.8)	316.0586 (78); 288.0635 (44); 273.0402 (13); 301.0350 (11); 245.0451 (10); 168.0058 (5)	314.0436 (100); 271.0244 (75); 283.2655 (41); 329.0687 (16); 299.0192 (11); 199.0383 (10)	L2a	[6]
3′,4′,7-Trihy-droxyflavanone	15,24	$C_{15}H_{12}O_5$	/	/	N.D	9574	271.0612 (+0)	/	135.0443 (100); 91.0195 (4);	L2a	[1]

									107.0498 (2); 153.0193 (1)		
Afzelin acetate isomer	15,29	$C_{23}H_{22}O_{11}$	$[M+Na]^+$ = 497.1063	4309	475.1243 (+1.7)	9611	473.1089 (-0.1)	171.0646 (100); 85.0276 (32); 189.0750 (24); 111.0437 (16); 129.0540 (14); 287.0540 (2)	284.0329 (100); 255.0303 (33); 473.1090 (30) 227.0352 (15); 413.0886 (6)	L2b	
alpha-Hy-droxy-dehydro-parain	15,5	$C_{21}H_{28}O_6$	$[M+Na]^+$ = 399.1789	4371	377.1962 (+0.9)	/	N.D	315.1594 (9); 283.1328 (9); 193.0858 (9); 165.0907 (8); 237.1282 (7); 271.1340 (7)	/	L2b	SI-RIUS
Flavonoid	15,6	$C_{16}H_{24}O_7$	$[M+NH_4]^+$ = 346.1861	4401	329.1596 (+0.4)	9819	327.145 (+0.2)	191.1076 (100); 209.1182 (83); 97.0291 (80); 145.1022 (67); 111.0449 (65); 163.1126 (57)	165.0924 (100); 150.0686 (2); 283.1908 (1)	L3	
Quassin	15,63	$C_{22}H_{28}O_6$	$[M+Na]^+$ = 411.1782	4413	389.1962 (+0.9)	/	N.D	223.0964 (100); 237.1122 (18); 177.0911 (17); 249.1122 (17); 208.0726 (14); 191.1069 (11)	/	L2a	[6]
Apigenin	15,64	$C_{15}H_{10}O_5$	/	4421	271.0602 (+0.4)	9864	269.0456 (+0.2)	271.0612 (100); 97.0292 (28); 153.0195 (27); 145.1021 (17); 191.1066 (11); 119.0496 (13)	269.0452 (100); 117.0344 (50); 151.0029 (12); 107.0138 (6); 225.0562 (5); 201.0563 (2)	L1	Stan-dard injec-tion
4-O-Acetylafze-lin	15,64	$C_{23}H_{22}O_{11}$	$[M+Na]^+$ = 497.1051	4424	475.1242 (+1.5)	9862	473.1089 (-0.1)	287.0546 (100); 129.0540 (34); 189.0751 (29); 111.0436 (15); 69.0328 (11); 83.0485 (7)	285.0394 (100); 473.1087 (66); 255.0297 (40); 227.0349 (26)	L2a	[1]
Neoquassin	15,77	$C_{22}H_{30}O_6$	$[M+Na]^+$ = 413.1934	4462	391.2118 (+0.7)	/	N.D	223.0969 (16); 285.1484 (7); 373.2020 (6);	/	L2b	[21]

								297.1491 (7); 207.1019 (5); 253.1590 (5)			
Kaempferol	15,88	$C_{15}H_{10}O_6$	$[2M-H]^-$ = 571.0882	4491	287.0552 (+0.6)	10015	285.0405 (+0.1)	287.0549 (100); 153.0180 (14); 121.0282 (7); 213.0543 (3); 165.0174 (3); 258.0530 (2)	117.0348 (3); 211.0400 (2); 143.0500 (3); 159.0449 (2); 227.0355 (2); 239.0352 (2)	L1	Standard injection
Nepetoidin B isomer	15,9	$C_{17}H_{14}O_6$	$[M+Na-2H]^-$ = 335.0534	4500	315.0864 (+0.3)	10020	313.0717 (-0.2)	163.0391 (100); 135.0440 (18); 145.0283 (7); 89.0380 (6); 123.0440 (6); 117.0337 (5)	161,0248 (100); 133,0298 (51); 151.0403 (11); 123.0457 (7); 105.0347 (2); 174.9564 (1)	L2a	[22]
Hispidulin	16,23	$C_{16}H_{12}O_6$	/	/	N.D	10203	299.0561 (+0)	/	284.0332 (100); 136.9887 (9); 227.0358 (4); 183.0459 (4); 117.0350 (4); 65.0040 (3)	L1	Standard injection
Zerumbone epoxide	16,48	$C_{15}H_{22}O_2$	$[M+Na]^+$= 257.1502	4667	235.1694 (+0.6)	/	N.D	123.1166 (100); 149.0953 (82); 81.0691 (62); 109.0649 (54); 91.0537 (53); 133.1010 (51)	/	L2b	[23]
Lignan (Acetoxypinoresinol derivative)	16,52	$C_{22}H_{24}O_8$	/	4674	417.1549 (+1.2)	10387	415.1401 (+0.6)	191.0712 (100); 181.0503 (51); 163.0761 (25); 167.0715 (24); 148.0531 (10); 131.0490 (9)	145.0287 (100); 415.1382 (21); 117.0359 (25); 321.8632 (10); 216.9194 (10); 195.9076 (9)	L3	

Lignan (Acetoxypino-resinol derivative)	16,65	$C_{21}H_{22}O_7$	/	4703	387.1442 (+1)	/	N.D	191.0705 (100); 151.0400 (47); 137.0604 (24); 163.0763 (21); 131.0492 (19); 148.0523 (12)	/	L3	
Zerumbone epoxide isomer	16,84	$C_{15}H_{22}O_2$	$[M+Na]^+$ = 257.1502	4727	235.1693 (+0.2)	/	N.D	151.1116 (100); 123.1164 (79); 109.0639 (43); 137.0959 (26); 81.0693 (25); 177.1271 (15)	/	L2b	
Nepetoidin B	16,95	$C_{17}H_{14}O_6$	/	4748	315.0863 (-0.1)	10542	313.0718 (+0.1)	163.0388 (100); 135.0438 (21); 117.0334 (12); 145.0276 (10); 89.0386 (7); 107.0495 (5)	161.0238 (100); 133.0289 (52); 151.0395 (11); 123.0446 (5); 105.0341 (3)	L2a	[22]
Cirsiliol	16,98	$C_{17}H_{14}O_7$	$[M+Na]^+$ = 353.0624	4762	331.0813 (+0.2)	10578	329.0668 (+0.4)	270.0514 (50); 298.0461 (19); 242.0565 (9); 316.0566 (9); 108.0201 (8); 154.0254 (7)	299.0198 (100); 314.0430 (67); 133.0295 (30); 199.0403 (23); 271.0251 (13); 243.0298 (8)	L1	Standard injection
Isokaempferide	17	$C_{16}H_{12}O_6$	$[M+Na]^+$ = 323.0525 $[M+Na+CO_2]^-$ = 367.0446	4776	301.0712 (+1.8)	10584	299.0562 (+0.3)	301.0701 (100); 286.0462 (69); 258.0516 (20); 121.0278 (8); 229.0488 (7); 213.0539 (6)	284.0328 (100); 227.0352 (75); 255.0300 (68); 183.0453 (6); 211.0401 (4)	L2a	[1]
Traumatic acid	17,21	$C_{12}H_{20}O_4$	$[M+Na]^+$ = 251.1265	4841	229.1436 (+0.7)	/	N.D	147.1180 (100); 123.1177 (92); 105.0707 (72); 81.0703 (64); 95.0861 (38); 119.0854 (34)	/	L2a	[6]
(10E,15E)-9,12,13-trihydroxyoctadeca-	17,34	$C_{18}H_{32}O_5$	$[2M-2H+Na]^-$ = 677.425	/	N.D	10739	327.2178 (+0.3)	/	211.1343 (74); 229.1449 (49); 171.1030 (28);	L2a	[1]

10,15-dienoic acid			$[M+Na+CO_2]^-$ = 395.20						291.1972 (11); 239.1296 (12); 221.1186 (11)		
2-[4-(5,7-dihydroxy-4-oxo-4H-chromen-2-yl)-2,6-dimethoxyphenoxy]-3-hydroxy-3-(4-hydroxy-3-methoxyphenyl)propyl acetate	17,41	$C_{29}H_{28}O_{12}$	$[M+Na]^+$ = 591.1477	4889	569.1664 (+1.8)	10755	567.1512 (+0.7)	359.0757 (100); 331.0804 (8); 316.0576 (2); 344.0555 (2); 539.1552 (2); 551.1572 (1)	357.0617 (100); 299.0234 (34); 180.9733 (29); 327.0139 (29); 294.9564 (23); 285.0430 (19)	L2b	
Pseudobaptigenin isomer	17,47	$C_{16}H_{10}O_5$	/	4903	283.0604 (+1.1)	10774	281.0456 (+0.2)	283.0600 (100); 225.0549 (49); 253.0495 (28); 169.0640 (13); 197.0608(11); 141.0697 (9)	281.0462 (100); 253.0508 (88); 195.0449 (16); 223.0396 (16); 209.0606 (10); 133.0300 (7)	L2b	[6]
Tryptanthrin	17,86	$C_{15}H_8N_2O_2$	$[M+Na]^+$ = 271.0476 $[2M+Na]^+$ = 519.1065	4980	249.0662 (+1.4)	/	N.D	130.0285 (39); 221.0710 (8); 78.0331 (3); 102.0337 (2); 166.0651 (2); 146.0230 (2)	/	L2a	[6]
Formononetin	148418	$C_{16}H_{12}O_4$	/	5023	269.0809 (+0.2)	11005	267.0663 (+0.1)	269.0806 (100); 226.0625 (10); 254.0576 (10); 197.0594 (9); 312.0896 (6); 118.0419 (5)	252.0426 (100); 223.0400 (42); 267.0659 (34); 195.0451 (33); 132.0215 (14); 135.0086 (10)	L2a	[6]
Sternbin	18,01	$C_{16}H_{14}O_6$	/	5030	303.0865 (+0.6)	11001	301.0717 (-0.2)	167.0338 (100); 163.0394 (97); 303.0862 (61); 135.0450 (34); 111.0441 (11); 145.0292 (9)	135.0448 (100); 165.0187 (22); 97.0296 (3); 149.9953 (2); 255.2341 (1)	L2a	[1]

Salvianolic acid C	18	$C_{26}H_{20}O_{10}$	/	5034	493.1134 (+1)	11019	491.0984 (+0.1)	313.0711 (100); 191.0352 (54); 123.0454 (23); 163.0386 (18); 160.0525 (16); 268.0758 (14)	179.0356 (100); 135.0455 (30); 161.0248 (24); 267.0678 (12); 311.0568 (8); 149.0247 (6)	L2a	[24]
Zerumbone epoxide isomer	18,35	$C_{15}H_{22}O_2$	/	5120	235.1685 (-3.2)	/	N.D	133.1011 (100); 105.0696 (90); 81.0695 (83); 123.0805 (67); 97.0654 (53); 179.1060 (50)	/	L2b	
5-hydroxy-6,7,8-trimethox-yflavone	18,78	$C_{18}H_{16}O_6$	/	5242	329.1023 (+1)	11330	327.0875 (+0.3)	329.1022 (100); 271.0599 (54); 296.0675 (34); 299.0550 (28); 330.1056 (22); 314.0791 (15)	297.0400 (100); 269.0449 (35); 254.0212 (31); 282.0163 (21); 136.9873 (18); 312.0631 (12)	L2b	SI-RIUS
Diacetylafzelin	18,87	$C_{25}H_{24}O_{12}$	$[M+Na]^+$ = 539.1166 $[M+Na+CO_2]^-$ = 583.1069	5276	517.1347 (+1.2)	11370	515.1197 (+0.4)	111.0439 (100); 83.0486 (76); 171.0651 (45); 287.0552 (37); 231.0866 (29); 129.0545 (13)	284.0328 (100); 255.0301 (24); 227.0353 (13)	L2a	[25]
Cirsimaritin	19,15	$C_{17}H_{14}O_6$	$[M+Na]^+$ = 337.0682	5360	315.0864 (+0.3)	11485	313.0719 (+0.4)	254.0574 (47); 282.0519 (18); 300.0643 (10); 108.0209 (10); 154.0264 (9); 136.0164 (9)	283.0251 (100); 298.0483 (53); 313.0716 (22); 255.0304 (20); 163.0039 (19); 117.0350 (19)	L2a	[1]
Jaceidin	19,67	$C_{18}H_{16}O_8$	/	/	N.D	11723	359.0771 (-0.4)	/	329.0310 (100); 344.0542 (79); 301.0372 (33); 273.0404 (18); 258.0172 (17); 217.0502 (17)	L2a	[1]

Pectolinarige-nin	19,74	$C_{17}H_{14}O_6$	/	5500	315.0863 (-0.1)	/	N.D	300.0633 (37); 272.0690 (18); 168.0060 (17); 186.0166 (12); 229.0502 (3); 257.0456 (3)	/	L2a	[6]
Eupatilin or iso-mer	19,74	$C_{18}H_{16}O_7$	/	5501	345.0971 (+0.6)	11760	343.0823 (-0.1)	345.0966 (100); 284.0678 (44); 312.0623 (17); 330.0731 (15); 148.0520 (8); 269.0443 (7)	328.0596 (100); 313.0359 (73); 298.0124 (51); 270.0172 (37); 343.0829 (25); 285.0406 (13)	L2b	[1]
Curcumenone	20,14	$C_{15}H_{22}O_2$	$[M+Na]^+$ = 257.1516	5611	235.1693 (+0.2)	/	N.D	93.0700 (100); 121.1010 (91); 105.0707 (54); 133.1011 (48); 161.0947 (34); 79.0540 (34)	/	L2a	[26]
Sakuranetin	20,27	$C_{16}H_{14}O_5$	/	5651	287.0914 (+0)	12015	285.0769 (+0.2)	123.1169 (100); 109.1012 (51); 287.2367 (38); 269.2259 (34); 173.1326 (32); 147.1170 (30)	119.0505 (100); 165.0201 (23); 283.2646 (19)	L2a	[1]
Cadinane ses-quiterpenoids	20,33	$C_{15}H_{24}O_2$	/	5664	237.1851 (+0.8)	/	N.D	105.0702 (100); 201.1637 (86); 219.1743 (79); 109.1016 (80); 95.0856 (79); 119.0860 (78)	/	L3	
Boronolide	20,36	$C_{18}H_{26}O_8$	$[M+Na]^+$ = 393.1520 $[M+NH_4]^+$ = 388.1967	5676	371.1702 (+0.4)	/	N.D	191.1065 (100); 209.1170 (68); 145.1009 (67); 97.0281 (51); 173.0960 (38); 163.1114 (37)	/	L2b	SIR-IUS
8-((1S,2S)-2-((S,E)-3-hy-droxypent-1-en-1-yl)-5-oxo-cyclopent-3-en-	20,56	$C_{18}H_{28}O_4$	$[M+Na]^+$ = 331.1878	5736	309.2063 (+0.8)	12153	307.1914 (-0.3)	291.1966 (00); 25.0968 (77); 273.1857 (72); 119.0859 (65); 97.1014 (61); 81.0704 (58)	185.1189 (100); 121.0660 (84); 235.1347 (75); 97.0660 (51);	L2b	

1-yl)octanoic acid									211.1343 (48); 125.0974 (40)		
Casticin	20,58	$C_{19}H_{18}O_8$	$[M+Na]^+$ = 397.0900	5745	375.1077 (+0.7)	12166	373.0929 (+0)	317.0655 (35); 299.0551 (20); 359.0760 (20); 345.0607 (11); 342.0731 (10); 327.0501 (7)	343.0463 (100); 358.0700 (67); 285.0042 (33); 300.0277 (29); 257.0092 (28); 328.0226 (15)	L1	Standard injection
Humulane sesquiterpene	20,61	$C_{15}H_{22}O_3$	$[M+Na]^+$ = 273.1465	5753	251.1645 (+1.3)	/	N.D	109.0648 (100); 175.1119 (33); 121.1013 (25); 93.0694 (18); 95.0852 (10); 105.0693 (8)	/	L3	
Indigo	20,66	$C_{16}H_{10}N_2O_2$	/	5782	263.0818 (+1.1)	/	N.D	235.0869 (16); 206.0839 (11); 132.0450 (12); 219.0924 (10); 180.0812 (5); 190.0647 (4)	/	L2a	[27]
Kaempferide (Kaempferol-4'-methyl ether)	20,92	$C_{16}H_{12}O_6$	/	5874	301.0715 (+2.8)	12345	299.0562 (-0.3)	301.0707 (100); 258.0526 (17); 230.0567 (16); 153.0180 (11); 286.0468 (8); 165.0189 (3)	284.0322 (100); 299.0562 (48); 151.0035 (15); 107.0137 (9); 164.0104 (7); 255.0297 (4)	L2a	[1]
3,7-Dihydroxy-3',4'-dimethoxyflavone (or isomer)	21,51	$C_{17}H_{14}O_6$	$[M+Na]^+$ = 337.0688	6067	315.0866 (+0.9)	12639	313.0718 (+0.1)	315.0867 (100); 300.0630 (58); 285.0392 (28); 257.0443 (22); 229.0492 (12); 272.0690 (7)	255.0300 (100); 283.0250 (89); 298.0483 (62); 313.0715 (18); 227.0347 (8); 183.0456 (7)	L2a	[1]
Bisdemethoxycurcumin	21,61	$C_{19}H_{16}O_4$	$[M+Na]^+$ = 331.0952	6108	309.1124 (+0.9)	12694	307.0977 (+0.4)	147.0439 (100); 225.0909 (38); 119.0492 (33); 131.0490 (11); 91.0541 (9); 107.0492 (8)	119.0500 (100); 143.0500 (26); 187.0394 (8); 93.0345 (1)	L2a	[1]

5-Hydroxy-2',4',7,8-tetramethoxyflavone	21,67	$C_{19}H_{18}O_7$	/	6135	359.1128 (+0.7)	/	N.D	359.1127 (100); 298.0836 (47); 326.0786 (18); 343.0814 (15); 162.0677 (12); 270.0885 (5)	/	L2a	[6]
Indirubin (Indigo Red)	21,73	$C_{16}H_{10}N_2O_2$	/	6156	263.0819 (+1.5)	12770	261.0669 (-0.2)	219.0921 (21); 235.0868 (16); 190.0658 (15); 206.0849 (9); 165.0705 (5); 132.0451 (5)	261.0670 (100); 157.0411 (68); 217.0768 (15); 233.0730 (6); 141.0477 (6); 185.0390 (2)	L2a	[28]
Trans-retinal isomer	21,87	$C_{20}H_{28}O$	/	6191	285.2212 (-0.3)	/	N.D	161.0965 (100); 285.2211 (56); 137.1328 (27); 95.0856 (20); 119.0857 (17); 105.0699 (16)	/	L2b	[29]
Demethoxycurcumin	21,99	$C_{20}H_{18}O_5$	$[M+Na]^+$ = 361.1057	6234	339.1230 (+0.9)	12890	337.1084 (+0.7)	147.0439 (100); 177.0544 (69); 255.1020 (62); 119.0490 (23); 223.0754 (15); 195.0804 (8)	119.0501 (100); 134.0371 (15); 173.0605 (14); 158.0370 (14); 143.0500 (11); 217.0508 (10)	L2a	[1]
Curcumin	22,36	$C_{21}H_{20}O_6$	$[M+Na]^+$= 391.1165	6362	369.1337 (+1.2)	13046	367.1187 (+0)	177.0547 (100); 145.0285 (50); 285.1125 (37); 117.0336 (13); 161.0596 (14); 151.0753 (12)	134.0371 (100); 149.0607 (55); 173.0606 (26); 158.0372 (23); 217.0510 (13); 201.0195 (5)	L1	Standard injection
Salvigenin	22,69	$C_{18}H_{16}O_6$	$[M+Na]^+$ = 351.0843	6489	329.1021 (+0.4)	/	N.D	329.1022 (100); 268.0732 (46); 296.0681 (17); 314.0784 (10); 133.0648 (9); 108.0209 (8)	/	L2a	[30]

Bisabolol	23,62	$C_{15}H_{26}O$	/	6778	223.2059 (+1.2)	/	N.D	149.1328 (100); 95.0859 (84); 135.1171 (70); 81.0695 (59); 109.1015 (51); 123.1172 (48)	/	L2b	SI-RIUS
Zerumbone	24,83	$C_{15}H_{22}O$	$[M+Na]^+$ = 241.1563	7187	219.1745 (+0.7)	/	N.D	81.0694 (100); 123.1168 (90); 109.0649 (63); 151.1117 (49); 95.0854 (36); 69.0694 (31)	/	L2a	[6,23]
7α-hydroxy-roylea-none(Hormi-none)	24,91	$C_{20}H_{28}O_4$	/	7221	333.2058 (-0.7)	14005	331.1915 (+0)	315.1958 (100); 220.1304 (43); 297.1841 (18); 95.0855 (16); 231.1036 (16); 171.0805 (15)	298.1573 (53); 270.1628 (13); 313.1806 (6); 285.1858 (6); 283.1344 (4); 243.1028 (2)	L2b	[31,32]
Caryophyllene oxide isomer	24,93	$C_{15}H_{24}O$	/	7229	$[M-H_2O + H]^+$ = 203.1793 (-0.6)	/	N.D	147.1171 (100); 109.1010 (99); 91.0542 (86); 203.1792 (83); 95.0854 (83); 105.0698 (82)	/	L2b	[6]
1-Palmito-ylphosphatidyl-choline	25,13	$C_{24}H_{50}NO_7P$	$[M+Na]^+$ = 518.3206	7292	496.3402 (+0.9)	/	N.D	184.0737 (100); 104.1074 (49); 86.0968 (9); 125.0002 (7); 313.2743 (4)	/	L2b	
Isolongifolol	25,18	$C_{15}H_{26}O$	/	7305	[M – H2O +H]$^+$= 205.195(-0.4)	/	N.D	121.1009 (100); 149.1324 (77); 107.0853 (77); 95.0852 (66); 109.1011 (65); 93.0695 (61)	/	L2a	[6]
Abetiane diterpenoid	25,29	$C_{20}H_{30}O$	/	7335	287.2369 (-0.1)	/	N.D	287.2363 (100); 163.1115 (83); 231.1739 (43); 109.1010 (36); 107.0853 (31); 123.1166 (22)	/	L3	

1-Hexadeca-noyl-sn-glyc-ero-3-phospho-(1'-myo-inosi-tol)	25,35	$C_{25}H_{49}O_{12}P$	/	7337	573.3034 (-0.1)	14171	571.289 (+0.2)	313.2735 (100); 155.0109 (20); 335.0734 (6); 393.2417 (5); 98.9840 (5); 239.2376 (5)	255.2333 (59); 241.0120 (25); 152.9961 (22); 315.0494 (18); 391.2241 (4); 78.9596 (4)	L2a	[1]
b-Eudesmol	25,59	$C_{15}H_{26}O$	/	7415	[M – H2O +H]+ = 205.1952 (+0.5)	/	N.D	95.0852 (100); 109.1012 (96); 81.0693 (28); 149.1324 (25); 135.1168 (23); 123.1167 (19)	/	L2a	[6]
Ibozol	25,72	$C_{20}H_{34}O_2$	[M+Na]+ = 329.2450 [2M+Na]+ = 635.5011	7457	[M+Na]+ = 329.245(+0.6)	/	N.D	271.2416 (82); 123.1166 (100); 109.1010 (71); 215.1793 (46); 81.0695 (36); 105.0696 (31)	/	L2b	[33]
b-Caryo-phyllene oxide	26,35	$C_{15}H_{24}O$	/	7632	[M – H_2O +H]+ = 203.1795 (+0.3)	/	N.D	105.0698 (100); 147.1166 (96); 203.1792 (90); 95.0853 (88); 119.0857 (87); 107.0852 (70)	/	L2a	[6]
12-Deacetoxy-scalaradial	27,54	$C_{25}H_{38}O_2$	/	7883	371.2945 (+0.1)	/	N.D	123.1170 (100); 269.2263 (55); 131.0857 (44); 199.1484 (26); 213.1640 (25); 81.0697 (24)	/	L2b	[34]
Retinol (Vita-min A)	30,1	$C_{20}H_{30}O$	/	8291	[M – H_2O +H]+ = 269.2259 (-1.7)	/	N.D	269.2261 (100); 239.1785 (75); 119.0850 (66); 254.2021 (52); 145.1008 (50); 123.1167 (39)	/	L2b	
Pheophorbide A	30,22	$C_{35}H_{36}N_4O_5$	[M+Na]+ = 615.2582	8313	593.2766 (+1.3)	/	N.D	533.2547 (48); 460.2251 (4); 565.2810 (3); 505.2235 (3); 447.2179 (2); 492.2516 (2)	/	L2a	[6]

Diacylglycerol	30,92	$C_{39}H_{64}O_5$	/	8459	613.4827 (+0.1)	/	N.D	613.4808 (100); 335.2572 (32); 259.2055 (28); 95.0858 (25); 109.1016 (22); 135.1172 (21)	/	L2b	

Table S4. Cytotoxic activity of 5 commercial compounds tested against two cancer cell lines

Compounds	CC_{50} [μM] [a]	
	RAW264.7	THP-1
(2) Cirsiliol	71.2 ± 44.1	11.2 ± 2.7
(3) Hispidulin	227.3 ± 36.7	21.1 ± 14.5
(5) α-bisabolol	174 ± 26.2	>250
(7) Zerumbone	44.5 ± 3.5	51.6 ± 31.6
(9) Pheophorbide A	20.9 ± 6.5	1.4 ± 0.8

[a] Experiments were performed as described in **Material and Methods** section. CC_{50}: Cytotoxic concentration leading to 50% death *in vitro* compared to the DMSO control. Data are expressed as mean ± standard deviation (n = 3).

Table S5. MZmine parameters. In red, parameters used in positive ionization mode. In blue, parameters used in negative ionization mode.

Module	Parameters
Raw data methods > Raw data import	Importation of all mzXML files
Raw data methods > Mass detection	Scans MS level: 1<br>Mass detector: centroid<br>Noise level: **1.0E3**; **3.0E2**
Raw data methods > Mass detection	Scans MS level: 2<br>Mass detector: centroid<br>Noise level: 1.5E1
Feature detection > LC-MS > ADAP chromatogram builder	Scans MS level: 1<br>Min consecutive scans: 3<br>Min intensity for consecutive scans: **3.0E3**; **9.0E2**<br>Min absolute height: **5.0E3**; **1.5E3**<br>Scan to scan tolerance (m/z): 10 ppm
Feature detection > Smoothing	Loess smoothing<br>Retention time width (scans): 3
Feature detection > Chromatogram resolving > Local minimum resolver	MS/MS scan pairing: checked<br>Retention time filter: use tolerance; 0,1 min (absolute)<br>Dimension: retention time<br>Chromatographic threshold: 85%<br>Minimum search range RT/Mobility (absolute): 0.05<br>Minimum relative height: /<br>Minimum absolute height: **3.0E3**; **1.0E3**<br>Min ratio of peak top/edge: 1.8<br>Peak duration range (min/mobility): 0.04 -1.00<br>Min scan data points: 3
Feature list methods > Isotopes > 13C isotope filter	m/z tolerance: 10 ppm<br>Retention time tolerance: 0.1 absolute (min)<br>Mobility tolerance: unchecked<br>Monotonic shape: unchecked<br>Maximum charge: 2<br>Representative isotope: most intense<br>Never remove feature with MS2: checked
Feature list methods > Alignment > Join aligner (within replicates)	m/z tolerance: 10 ppm<br>Weight for m/z: 75<br>Retention time tolerance: 0.2 absolute (min)<br>Weight for RT: 25<br>Mobility tolerance: unchecked<br>Mobility weight: 1.00<br>Require same charge state: checked<br>Require same ID: unchecked<br>Compare isotope pattern: unchecked<br>Compare spectra similarity: unchecked
Feature list methods > Feature list filtering > Feature list rows filter (within replicates)	Minimum aligned features (samples): 2 for extracts and fractions; 3 for blanks and parabens<br>Retention time: 0.6 - 31.0 min (auto range)<br>Features with MS2 scans: unchecked

Feature list methods > Alignment > Join aligner (with blanks and parabens)	m/z tolerance: 10 ppm<br>Weight for m/z: 75<br>Retention time tolerance: 0.2 absolute (min)<br>Weight for RT: 25<br>Mobility tolerance: unchecked<br>Mobility weight: 1.00<br>Require same charge state: checked<br>Require same ID: unchecked<br>Compare isotope pattern: unchecked<br>Compare spectra similarity: unchecked
Feature list methods > Alignment > Join aligner (across all samples)	m/z tolerance: 10 ppm<br>Weight for m/z: 75<br>Retention time tolerance: 0.2 absolute (min)<br>Weight for RT: 25<br>Mobility tolerance: unchecked<br>Mobility weight: 1.0<br>Require same charge state: checked<br>Require same ID: unchecked<br>Compare isotope pattern: unchecked<br>Compare spectra similarity: unchecked
Feature list method > Feature list filtering > Feature list rows filter (across all samples)	Minimum features in a row (abs or %): unchecked<br>Retention time: 0.6 - 31.0 min (auto range)<br>Features with MS2 scans: checked<br>Reset the feature number ID: checked
Feature list method > Export feature list > GNPS - feature based molecular networking	Merge MS/MS (experimental): checked<br>Select spectra to merge: across samples<br>m/z merge mode: weighted average (remove outliers)<br>Intensity merge mode: sum intensities<br>Expected mass deviation: 5 ppm<br>Cosine threshold: 70 %<br>Signal count threshold: 20 %<br>Isolation window offset: 0.0<br>Isolation window width (m/z): 3.0<br>Filter rows: ALL<br>Feature intensity: Peak area<br>CSV export: simple<br>Submit to GNPS: unchecked<br>Open folder: unchecked

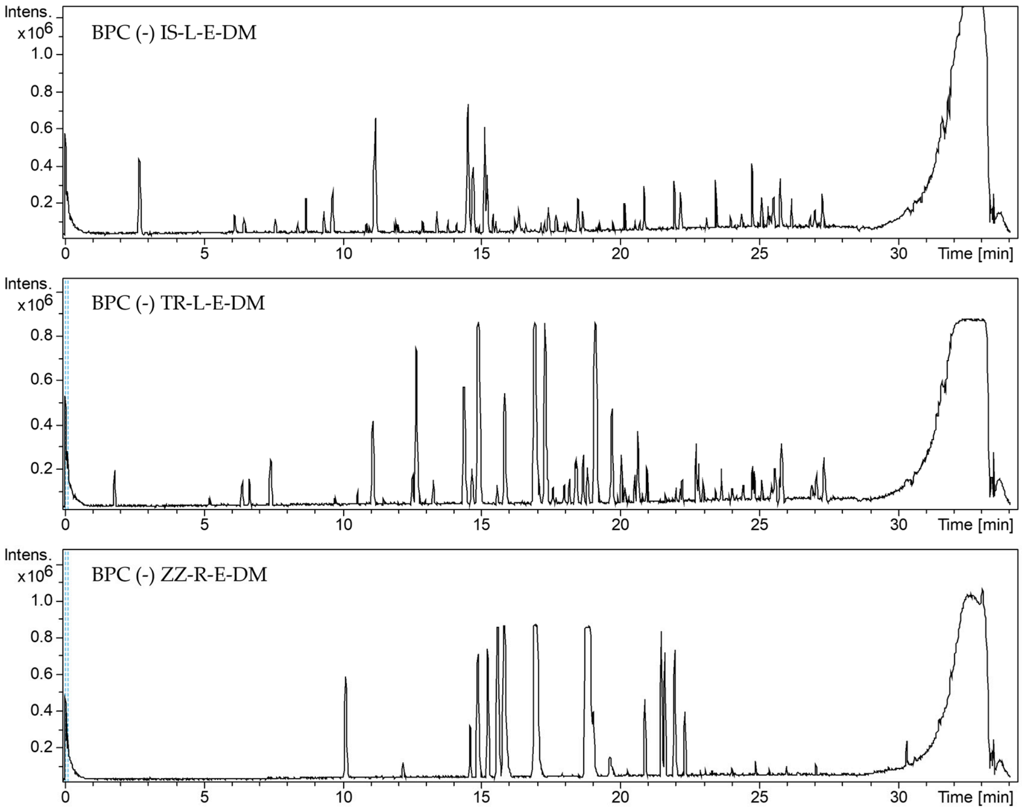

Figure S2. Base Peak Chromatograms (BPCs), acquired in negative ionization mode, of some active dichloromethane fractions.

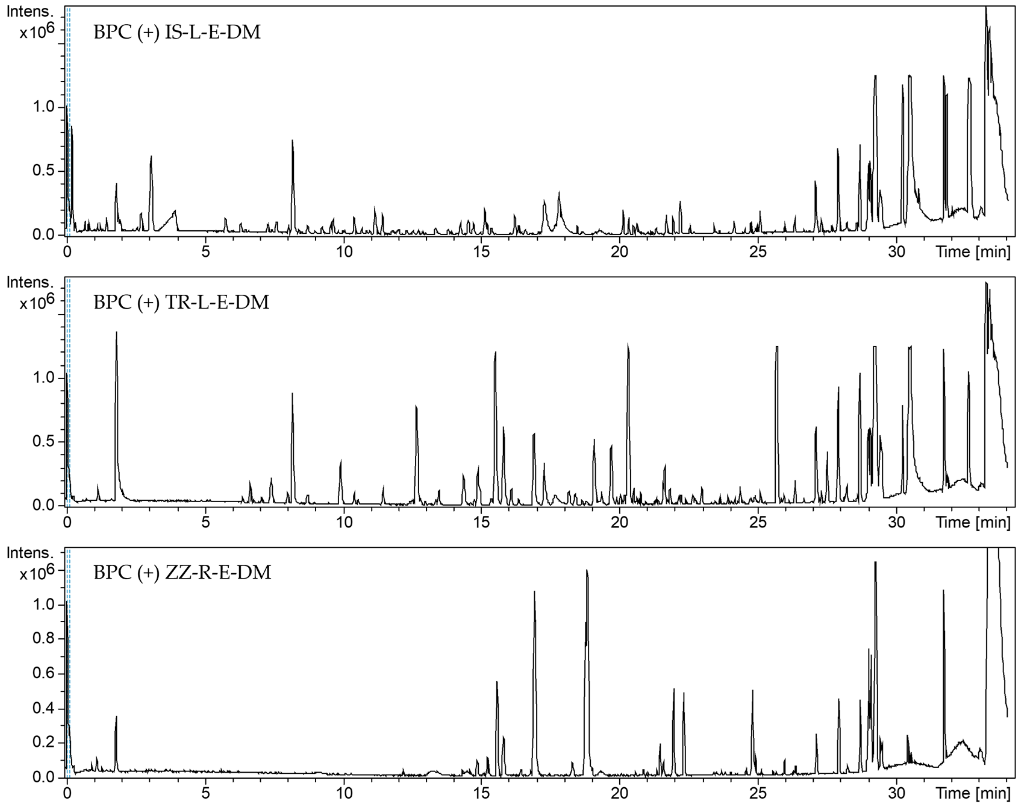

Figure S3. Base Peak Chromatograms (BPCs), acquired in positive ionization mode, of some active dichloromethane fractions.

Abbreviations

The following abbreviations are used in this manuscript:

MDPI	Multidisciplinary Digital Publishing Institute
DOAJ	Directory of open access journals
TB	Tuberculosis
Mtb	*Mycobacterium tuberculosis*
NPs	Natural Products
MN	Molecular Network
MS	Mass Spectrometry
GNPS	Global Natural Products Social Molecular Networking

References
1. GNPS Job - Negative Ionization Mode.
2. A Study of the Fragmentation of Caprolactam and Its Methyl Derivatives under Electron-impact - Mitera - 1971 - Organic Mass Spectrometry - Wiley Online Library Available online: https://analyticalsciencejournals-onlinelibrary-wiley-com.lama.univ-amu.fr/doi/abs/10.1002/oms.1210050604 (accessed on 17 July 2025).
3. Djoumbou-Feunang, Y.; Pon, A.; Karu, N.; Zheng, J.; Li, C.; Arndt, D.; Gautam, M.; Allen, F.; Wishart, D.S. CFM-ID 3.0: Significantly Improved ESI-MS/MS Prediction and Compound Identification. *Metabolites* **2019**, *9*, 72, doi:10.3390/metabo9040072.
4. MoNA Spectrum 023897 - Splash10-001i-0900000000-B32205397e33e164786f.
5. Singh, A.; Kumar, S.; Kumar, B. LC-MS Identification of Proanthocyanidins in Bark and Fruit of Six Terminalia Species. *Natural Product Communications* **2018**, *13*, 1934578X1801300511, doi:10.1177/1934578X1801300511.
6. GNPS Job - Positive Ionization Mode.
7. Breaud, C.; Lallemand, L.; Mares, G.; Mabrouki, F.; Bertolotti, M.; Simmler, C.; Greff, S.; Mauduit, M.; Herbette, G.; Garayev, E.; et al. LC-MS Based Phytochemical Profiling towards the Identification of Antioxidant Markers in Some Endemic Aloe Species from Mascarene Islands. *Antioxidants (Basel)* **2022**, *12*, 50, doi:10.3390/antiox12010050.
8. MassBank Spectrum - Isatin -MSBNK-Fiocruz-FIO00511.
9. MassBank Spectrum - Indole-3-Carboxyaldehyde - MSBNK-RIKEN-PR101043.
10. Massbank Spectrum - Flavonol Base + 3O, O-dHex, O-Hex-Hex - MSBNK-RIKEN-PR309263.
11. Massbank Spectrum - Catechin Gallate -MSBNK-BS-BS003891.
12. Massbank Spectrum - Ditolylguanidine - MSBNK-MSSJ-MSJ02138.
13. Winstel, D.; Marchal, A. Lignans in Spirits: Chemical Diversity, Quantification, and Sensory Impact of (±)-Lyoniresinol. *Molecules* **2019**, *24*, 117, doi:10.3390/molecules24010117.
14. MassBank Spectrum - Quercetin-3-O-Glucoside - MSBNK-RIKEN-PR040186.
15. Massbank Spectrum - Luteolin-7-O-Glucoside - MSBNK-RIKEN-PR305600.
16. Puyvelde, L.V.; Kimpe, N.D.; Dubé, S.; Chagnon-Dubé, M.; Boily, Y.; Borremans, F.; Schamp, N.; Anteunis, M.J.O. 1′,2′-Dideacetylboronolide, an α-Pyrone from *Iboza Riparia*. *Phytochemistry* **1981**, *20*, 2753–2755, doi:10.1016/0031-9422(81)85280-6.
17. Massbank Spectrum - Flavonol Base + 4O, O-MalonylHex - MSBNK-RIKEN-PR310913.
18. Massbank Spectrum - 3-O-Neohesperidoside Kaempferol - MSBNK-BS-BS001029.
19. Massbank Spectrum - 6-Beta-Hydroxycortisol - MSBNK-Athens_Univ-AU283802.
20. MassBank Spectrum - Eriodictyol - MSBNK-RIKEN-PR306152.
21. Barbetti, P.; Grandolini, G.; Fardella, G.; Chiappini, I. Quassinoids from Quassia Amara. *Phytochemistry* **1993**, *32*, 1007–1013, doi:10.1016/0031-9422(93)85245-M.
22. Kim, M.; Kim, J.Y.; Yang, H.S.; Choe, J.-S.; Hwang, I.G. Nepetoidin B from Salvia Plebeia R. Br. Inhibits Inflammation by Modulating the NF-κB and Nrf2/HO-1 Signaling Pathways in Macrophage Cells. *Antioxidants (Basel)* **2021**, *10*, 1208, doi:10.3390/antiox10081208.
23. Dai, J.-R.; Cardellina, J.H.; Mahon, J.B.M.; Boyd, M.R. Zerumbone, an HIV-Inhibitory and Cytotoxic Sesquiterpene of Zingiber Aromaticum and Z. Zerumbet. *Natural Product Letters* **1997**, *10*, 115–118, doi:10.1080/10575639708043725.
24. Achard-Baccati, C.; Garayev, E.; Saïd Hassane, C.; Breaud, C.; Garaev, E.; Bertolotti, M.; Mabrouki, F.; Bun-Llopet, S.-S.; Baghdikian, B. Can Provence Flora Offer Effective Alternatives to Widely Used Medicinal Plants? A Comparative Study of Antioxidant Activity and Chemical Composition Using Molecular Networking. *Molecules* **2025**, *30*, 2072, doi:10.3390/molecules30092072.

25. Nakatani, N.; Jitoe, A.; Masuda, T.; Yonemori, S. Flavonoid Constituents of Zingiber Zerumbet Smith. *Agricultural and Biological Chemistry* **1991**, *55*, 445–460, doi:10.1080/00021369.1991.10870577.
26. Liu, F.; Bai, X.; Yang, F.-Q.; Zhang, X.-J.; Hu, Y.; Li, P.; Wan, J.-B. Discriminating from Species of Curcumae Radix (Yujin) by a UHPLC/Q-TOFMS-Based Metabolomics Approach. *Chinese Medicine* **2016**, *11*, 21, doi:10.1186/s13020-016-0095-8.
27. Eugenia, C. Indigofera species, source of indigo - the most valuable and used natural "dye" in the world.
28. MoNa Spectrum - Indirubin - Https://Mona.Fiehnlab.Ucdavis.Edu/Spectra/Browse?Query=exists(Splash.Splash:%27splash10-03di-0090000000-5ac471605d8d39b4e191%27).
29. MassBank Spectrum - All Trans-Retinal - MSBNK-BGC_Munich-RP014201.
30. MassBank Spectrum - Salvigenin - MSBNK-Univ_Toyama-TY000249.
31. Baldin, V.P.; Scodro, R.B. de L.; Lopes-Ortiz, M.A.; de Almeida, A.L.; Gazim, Z.C.; Ferarrese, L.; Faiões, V. dos S.; Torres-Santos, E.C.; Pires, C.T.A.; Caleffi-Ferracioli, K.R.; et al. Anti-Mycobacterium Tuberculosis Activity of Essential Oil and 6,7-Dehydroroyleanone Isolated from Leaves of Tetradenia Riparia (Hochst.) Codd (Lamiaceae). *Phytomedicine* **2018**, *47*, 34–39, doi:10.1016/j.phymed.2018.04.043.
32. Allen, F.; Greiner, R.; Wishart, D. Competitive Fragmentation Modeling of ESI-MS/MS Spectra for Putative Metabolite Identification. *Metabolomics* **2015**, *11*, 98–110, doi:10.1007/s11306-014-0676-4.
33. Zelnik, R.; Rabenhorst, E.; Matida, A.K.; Gottlieb, H.E.; Lavie, D.; Panizza, S. Ibozol, a New Diterpenoid from Iboza Riparia. *Phytochemistry* **1978**, *17*, 1795–1797, doi:10.1016/S0031-9422(00)88701-4.
34. Van Puyvelde, L.; Nyirankuliza, S.; Panebianco, R.; Boily, Y.; Geizer, I.; Sebikali, B.; De Kimpe, N.; Schamp, N. Active Principles of Tetradenia Riparia. I. Antimicrobial Activity of 8(14),15-Sandaracopimaradiene-7α,18-Diol. *Journal of Ethnopharmacology* **1986**, *17*, 269–275, doi:10.1016/0378-8741(86)90115-7.
